# Communication With Patients Before an Operation: Their Preferences on Method of Communication

**DOI:** 10.7759/cureus.11431

**Published:** 2020-11-11

**Authors:** Abdallah Al Ghunimat, Jamie Hind, Amr Abouelela, Gur Aziz Singh Sidhu, Andrew Lacon, Neil Ashwood

**Affiliations:** 1 Trauma and Orthopaedics, University Hospitals of Derby and Burton NHS Foundation Trust, Burton-on-Trent, GBR

**Keywords:** communication in healthcare, patients satisfaction, orthopaedics surgery, better outcomes, technology

## Abstract

Background

With the constantly evolving communication technologies, it is essential for all healthcare professionals to try utilising various methods in communicating with patients. This will lead to better healthcare outcomes and patient satisfaction.

Objective

The aim of the study was to compare a patient’s preference to various communication methods regarding their appointments and to evaluate if we’re giving our patients an appropriate notice period prior to their operation.

Methods

A questionnaire was given to 111 patients who underwent elective orthopaedic procedures.

Results

Factors like age and gender affect the choice of communication method. Traditional letters still have a role for an older population aged 65 and over. However, younger patients showed higher preference for other communication methods such as phone calls, texts, and e-mails. Gender also had a role in choosing a preference where male patients chose a range of options whilst female patients preferred phone calls. Most patients stated they received an appropriate notice period, with 88% of patients stating they would like to be notified one-two weeks prior to their operation.

Conclusion

More research needs to be conducted into using text messages and e-mails in communicating with elective surgical patients, in addition to implementing newer technologies like mobile phone applications and secure online messaging portals, as this has the potential to reshape the communication process with our patients and lead to better health outcomes and patient satisfaction.

## Introduction

Communicating with patients prior to their operation is a key element in preparation for the surgery they are about to have. It is fundamental that patients are given appropriate times, relevant information, and guidance about their operation, in addition to clear and concise instructions about what to do on the day itself. Communication must be done in a timely manner in order for patients to prepare and manage their schedule to fit around their operation.

As technology advances, the method of communication has evolved into a variety of options keeping in sync with modern-day technology. As opposed to 10 years ago, postal communication may not be the ideal preference anymore as people now communicate via electronic mail, or using mobile devices. Instead of phone calls, texting may be the ideal and more convenient way of communicating with patients. Therefore, it is important to regularly familiarize ourselves on how patients would like to be informed preoperatively, and ideally give them a range of options for their mode of preference.

Age of the patient has a role in choosing the preferred communication method; in a study carried out asking how patients wanted to be contacted regarding surveys, favouring only letter contact increased substantially with age [1].

With the increasing use of mobile phones, introducing text messages as a mean of communication can have a positive impact on patient’s satisfaction. Patients perceived this form of communication as useful and felt that it kept them better informed [2]. In a literature review conducted over studies evaluating the use of mobile phone text messages in health care services, it was found that 77% (46/60) of the studies showed improved outcomes [3].

When it comes to using newer technologies in the communications process such as e-mails, one study by Neill et al. showed that 85% of patients believed e-mail would be a good way for a patient to communicate with his/her physician [4]. Moreover communicating by e-mail can clarify advice previously given and point patients towards information materials and other resources available on the internet [5].

## Materials and methods

This is a prospective study involving 111 patients aged 17-88. An anonymous questionnaire was given to patients who are undergoing elective orthopaedic surgeries, asking what would be the best way to communicate with them prior to their surgical appointments.

Methods of communication included conventional letters, emails, phone calls, and phone texts. Patients could choose more than one preferred communication method. We also enquired about preferred notice period for them to be contacted prior to their surgery date, whether they received a confirmation letter, and overall satisfaction with the communication process.

## Results

**Figure 1:**
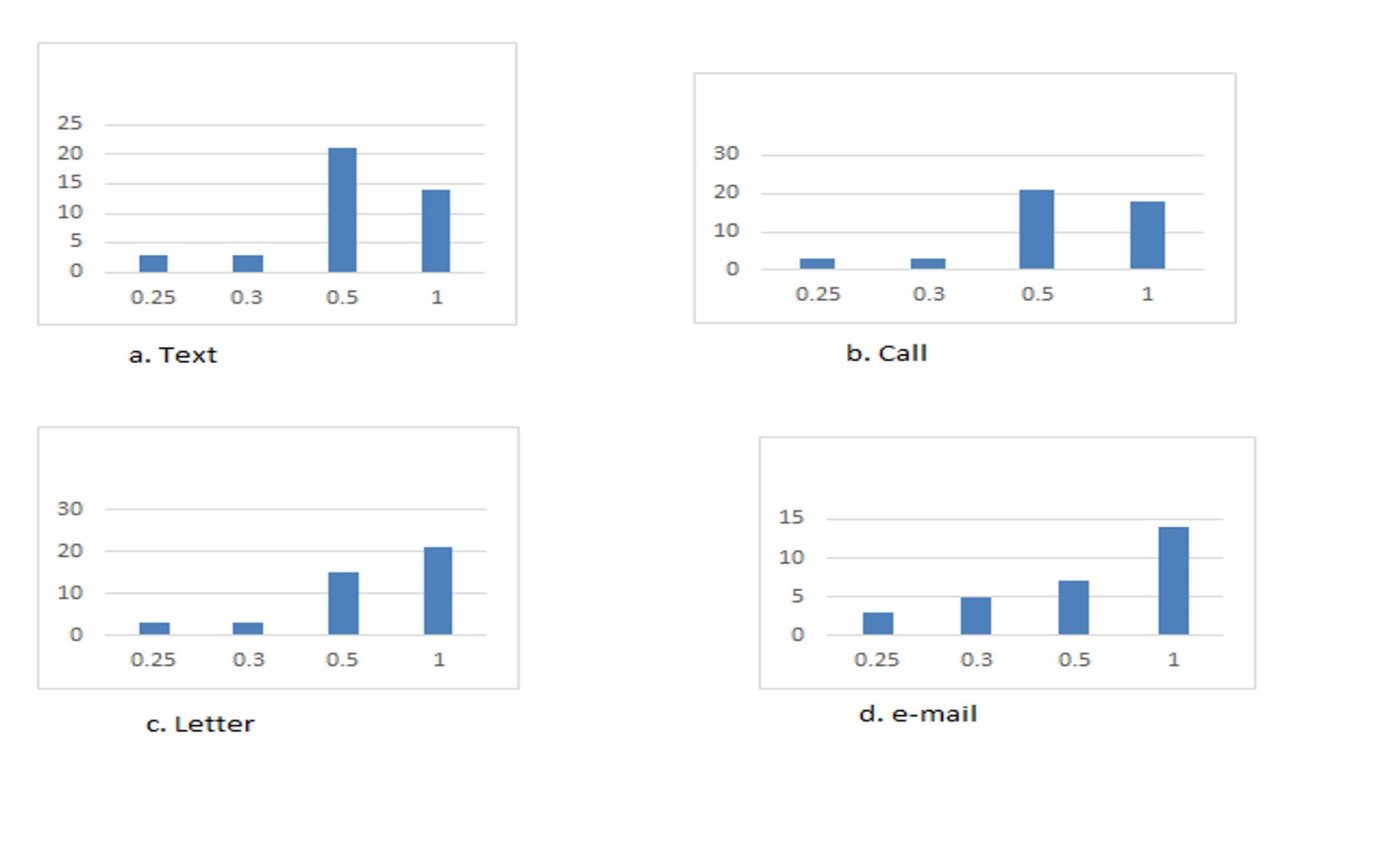
Bar charts demonstrating patients' preference across different communication methods. A: Text, B: Phone call, C: Letter, D: E-mail

Overall, keeping in mind patients had the option to choose more than one method, phone calls were the most popular with 45 patients choosing this as a preference. The second most preferred choice was an equal number between texts and letters with 44 each. The least preferred choice was communication by e-mails with 30 patients opting for this method. Among the 111 patients, 69 patients chose only one method as a preference; 21 by letter, 18 by phone calls, and 15 for texts and e-mails each (Figure 1).

**Figure 2:**
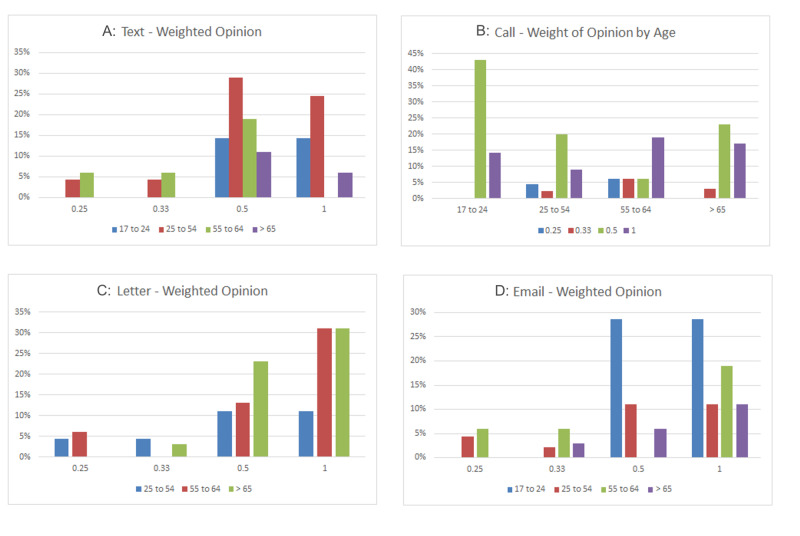
Bar charts demonstrating weighted opinion for every communication method in different age groups. A: Text, B: Phone call, C: Letter, D: E-mail

Looking more closely into trends according to age groups, patients aged 17-24 favored phone calls and e-mails followed by texts, and conventional letters were not wanted. In the prime working-age group (25-54), all modes of communication were welcomed, with texts and e-mails being the most popular, followed by phone calls and letters closely. Mature working patients aged 55-64 showed a higher preference for letters, where the rest of the options were valued equally. Elderly people aged 65 and over favored letters followed by phone calls (Figure 2).

**Figure 3:**
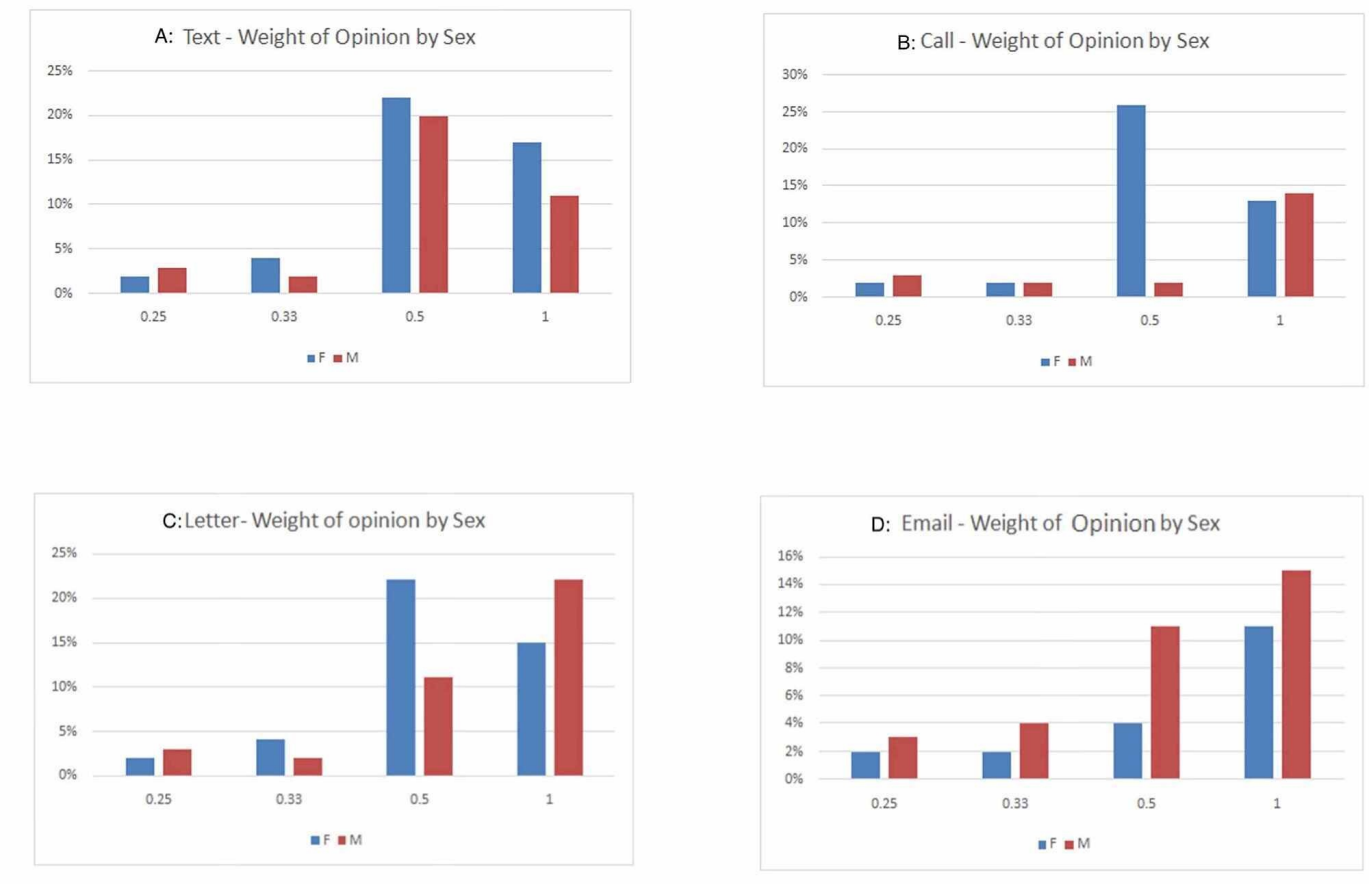
Bar charts demonstrating weighted opinion by gender for different communication methods. A: Text, B: Phone call, C: Letter, D: E-mail

Gender wise, the study included 46 female and 65 male patients. Amongst females, we found a slight preference for text messages, followed closely by phone calls and letters, whereas e-mails were not very favorable. On the other hand, male patients preferred phone calls and letters over texts and e-mails (Figure 3).

**Figure 4:**
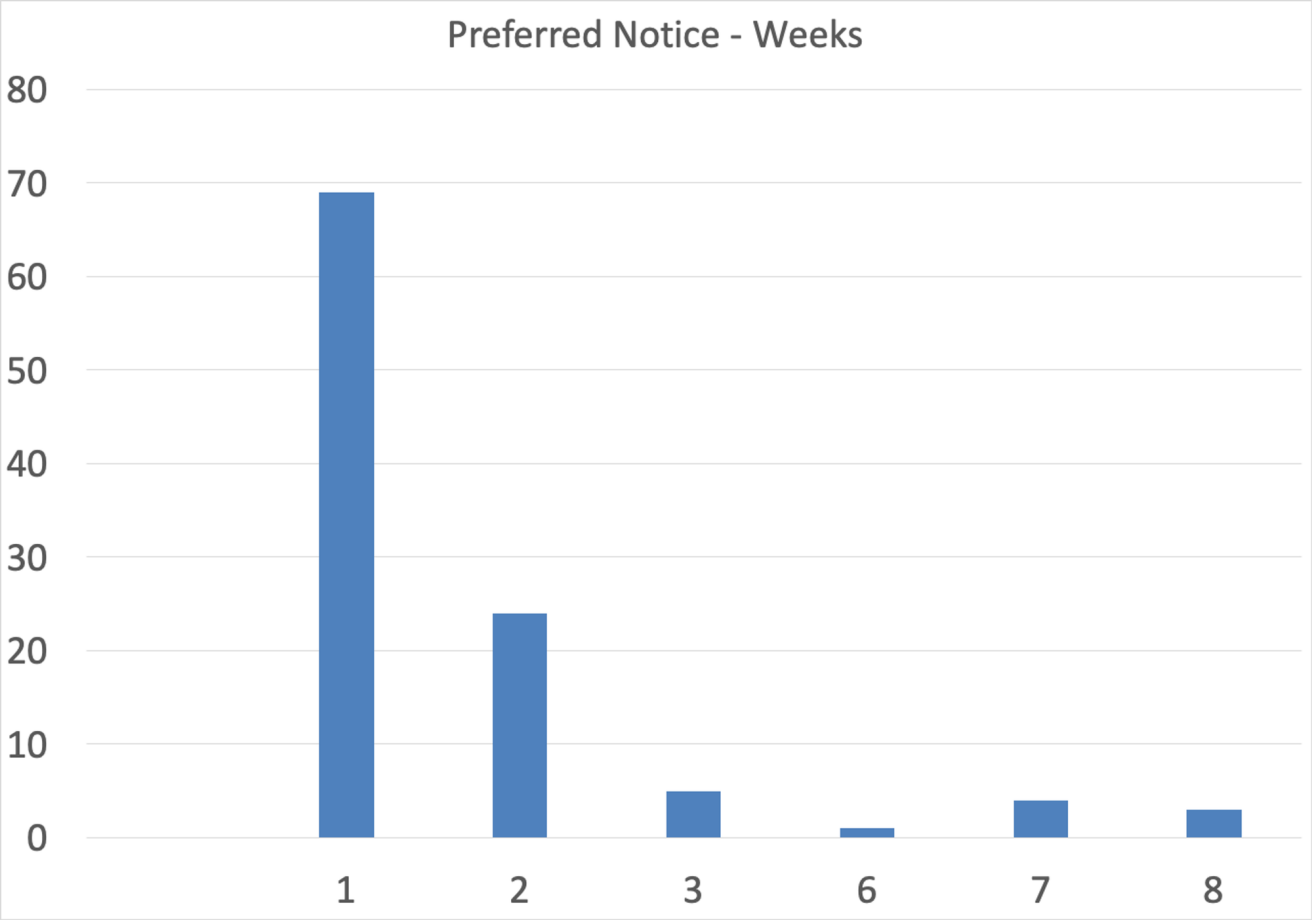
Bar chart demonstrating the percentage of patients (Y-axis) in terms of preferred notice period prior to surgery in weeks (X-axis).

When it came to notice before surgery, most patients were notified 1-5 days before their surgery, with the younger working-age group 17-24 given more notice period at 6-10 days. The majority of patients stated they would prefer at least one-week notice before their appointment, with females asking for a slightly longer notice period of one-two weeks. Receipt of confirmation letters was approved mostly by the elderly group > 65 and the least by group aged 17-24 (Figure 4)​​​​​​.

At the end of the survey, patients were asked to rank their overall satisfaction with the communication process on a scale of 0%-100%. The vast majority of the group 94.5% was highly satisfied (80%-100%). The young age group 17-24 was the most satisfied, while elderly patients were the least.

## Discussion

Effective and clear communication is essential to provide the best care possible for patients. Communication with surgical patients has to be even more clear and precise as most of the time the surgeon is delivering a large number of instructions and education ahead of anticipated surgery. Hence it is important for surgeons to develop skills that enhance patient education and counselling as this will lead to improving patient recall of information, compliance, satisfaction, and psychological well-being [6].

It’s worth taking time to evaluate the whole communication process with surgical patients, from the moment of the decision to undergo surgery is taken to the post-operative follow-up. Communicating with patients has a big impact on managing the surgical list, and using more modern communication methods may have a positive role in managing those lists. As the decision to perform an elective operation usually takes place during an outpatient visit, offering a date for surgery on that visit, or pre-booking may prove to be more time-efficient for both the surgeon and the patient, keeping in mind having a solid communication platform will support this system going forwards, as both parties can get in touch quicker and more efficiently making sure no valuable theatre slots are lost. Making use of such a system can improve cancellation rates [7], one study suggested that in the case of pre-booking, cancellation of high-priority elective procedures was only one-third as likely as it was in the case of booking from waitlists [8]. One of the common causes of surgery cancellations is inconvenient appointments [9], which can be overcome by choosing a date with the patient during the outpatient consultation.

Nowadays with almost everybody owning a mobile phone, people are accessible almost all the time and it does not have to be with a phone call. Using text messages can be more effective and is certainly quicker than sending a conventional letter. It can be structured to deliver important information and reminders both preoperatively and postoperatively, patients found the information useful, easy to understand, and it also helped reduce anxiety or level of worry during and after surgery [10]. Moreover, it can increase adherence to medications and clinic attendance while on the other hand decrease emergency room visits and readmissions [11]. It’s a low-cost way of delivering information when needed to patients and can improve their satisfaction with the whole communication process [12]. With the rapidly growing use of smartphones, an application could be utilized to facilitate communication with patients and overall medical care. This can offer a broad spectrum of benefits such as appointment booking, behavioural modifications, patient monitoring, and adherence to medical protocols [13].

More than ever before people are spending time on the internet whether to seek information or to get in touch with each other, e-mails can be accessed anywhere using computers, laptops, smart tablets or now through most mobile phones enabled with internet access, so keeping that in mind using e-mails as a mean of communication can be a powerful tool and has the potential to significantly improve communication prior to elective surgeries, leading to an increase in patient’s satisfaction [14,15]. This can offer many advantages such as time-saving, and when compared to a phone call, it offers patients the chance to read the e-mail at a convenient time when they are busy and not able to answer the phone, additionally, an e-mail is a better way of documentation in contrast of writing in the records that a phone call was made [16]. Moreover, e-mail communication offers increased access to the care provider and more comfort in asking questions, as some patients were observed to open up more than in face to face consultations [16,17]. Despite the perception that using emails is solely for younger age groups, offering a better communication process through e-mails was met by enthusiasm in some patients over the age of 65, although this decreased as age goes up [18]. One of the potentials with using this platform is attaching links to online available information resources, which can be invaluable to a patient before going ahead with a procedure and can eliminate some of the associated anxiety, in addition to having access to postoperative instructions and answers to frequent questions related to their surgery [16].

Some disadvantages and potential risks were identified with the use of e-mails, amongst those were privacy and confidentiality issues [16], especially when there aren’t enough guidelines on the subject [14]. One of the widely proposed solutions to overcome such concerns is using secure online messages in a carefully designed patient-doctor portal [17,19,20].

## Conclusions

Gender and age influence the preferred mode of communication. A wider range of communication (texts, emails, and letters) are preferred by men, women seem to prefer phone calls. The number of days’ notice provided by a mode of communication influences the weighted opinion given. Those who prefer letters are those who obtained them within one to five days of their appointment. Of those who prefer emails and calls, most did not receive a letter or obtained slightly less notice. Letters still have a positive role in notifying patients greater than 65 years, however, there are a significant number not obtaining letters and would prefer a different mode of communication. Almost all patients stated they have received appropriate notice period, with one-two weeks being the period favored by the most. Majority of participants were satisfied overall. More research needs to be conducted into using text messages and e-mails in communicating with elective surgical patients, in addition to implementing newer technologies like mobile phone applications and secure online messaging portals, as this has the potential to reshape the communication process with our patients and lead to better health outcomes and patient satisfaction.
